# Preterm Birth and Its Long-Term Effects: Methylation to Mechanisms

**DOI:** 10.3390/biology3030498

**Published:** 2014-08-21

**Authors:** Sasha E. Parets, Carrie E. Bedient, Ramkumar Menon, Alicia K. Smith

**Affiliations:** 1 Genetics and Molecular Biology Program, Emory University, Atlanta, GA 30322, USA; E-Mail: sparets@emory.edu; 2 Department of Gynecology & Obstetrics, Emory University School of Medicine, Atlanta, GA 30322, USA; E-Mail: carrie.bedient@emory.edu; 3 Division of Maternal-Fetal Medicine Perinatal Research, Department of Obstetrics & Gynecology, The University of Texas Medical Branch at Galveston, Galveston, TX 77555, USA; E-Mail: ra2menon@UTMB.edu; 4 The Perinatal Research Center, Nashville, TN 37203, USA; 5 Department of Psychiatry & Behavioral Sciences, Emory University School of Medicine, Atlanta, GA 30322, USA

**Keywords:** DNA methylation, preterm birth, pregnancy, DOHaD, epigenetic, developmental programming, gestational age

## Abstract

The epigenetic patterns established during development may influence gene expression over a lifetime and increase susceptibility to chronic disease. Being born preterm (<37 weeks of gestation) is associated with increased risk mortality and morbidity from birth until adulthood. This brief review explores the potential role of DNA methylation in preterm birth (PTB) and its possible long-term consequences and provides an overview of the physiological processes central to PTB and recent DNA methylation studies of PTB.

## 1. Introduction

Despite advances in healthcare, preterm birth (birth prior to 37 weeks gestation) remains a major global health problem [1]. Preterm birth increases risk for morbidity and mortality in the first year of life [2]. The consequences of preterm birth extend throughout development. For example, children born preterm have higher levels of neurodevelopmental disability and an increased risk of behavioral problems such as Attention Deficit Hyperactivity Disorder [3,4,5]. Being born preterm also increases risk for developing chronic diseases such as hypertension, type 2 diabetes, cardiovascular disease, obesity and psychiatric disorders [2,6,7]. Collectively, these observations support the developmental origin of health and disease (DOHaD) hypothesis, which conceptually links the prenatal and early postnatal environments to the development of chronic diseases [8,9].

**Figure 1:**
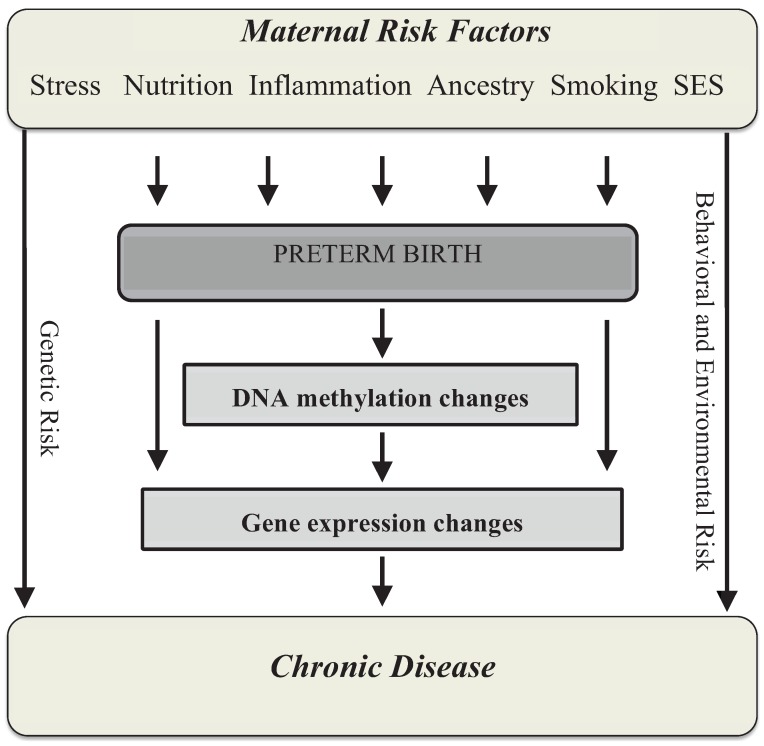
A model for the involvement of DNA methylation in the development of chronic disease following preterm birth (PTB). Multiple maternal risk factors can increase risk of PTB through independent biological mechanisms that may produce changes in DNA methylation or other epigenetic mechanisms. Such risk factors include, but are not limited to, stress, nutrition, immune conditions that produce inflammation, ancestry, smoking and socioeconomic status (SES). These epigenetic changes influence gene expression and thus the developmental trajectory of the neonate. Risk of developing chronic diseases may also be influenced by genetic predisposition and independent behavioral or environmental factors.

The molecular mechanisms that underlie the relationship between preterm birth and its developmental consequences are not clear, but advocates of the DOHaD hypothesis believe that epigenetics may play a key role (Figure 1) [10]. Epigenetic modifications induce changes in gene expression through structural alterations of DNA that are maintained through each round of cell division; they respond to changes in the environment, are potentially reversible and can be targeted for disease therapies [11]. DNA methylation at cytosine-guanine dinucleotides (CpG sites), the most commonly studied epigenetic modification in humans, guides temporal and tissue-specific gene expression during fetal development and tissue differentiation [12]. Subtle differences in the intrauterine environment may influence this tightly controlled process such that environmentally-induced epigenetic changes may result in stable phenotypic differences. This review will provide an overview of the key physiological features of pregnancy and spontaneous preterm birth, a major subset of all preterm births with unknown etiology, with specific emphasis on the emerging role of DNA methylation in the field.

This is not meant to serve as a comprehensive review of preterm birth. Other excellent reviews of its epidemiology, management, prediction, prevention, physiology, and proposed mechanisms have recently been published [2,3,4,13,14,15,16,17,18]. Instead, this brief review will highlight the paucity of DNA methylation studies in the field and the opportunities for using DNA methylation studies to advance our understanding of the causes and consequences of preterm birth.

## 2. Overview of Pregnancy

Pregnancy initiates after fertilization of the female gamete and implantation of the embryo; it is the gestational period during which fetal development and growth occurs. The typical duration of human pregnancy is 280 days (40 weeks), though the actual length may vary considerably [19,20]. Obstetricians calculate the estimated due date of a pregnancy starting from the first day of the last menstrual period (LMP) [21]. The LMP marks the beginning of the first trimester, which is characterized by embryonic implantation, organogenesis and maternal adaptations of the cardiovascular, respiratory, renal, endocrine and immune systems [22,23]. These changes are maintained during the second and third trimesters to support rapid growth and development of the fetus.

The relationship between the mother and fetus is considered to be semi-allografic because the fetus is genetically distinct and can be recognized as a foreign body by the maternal immune system. The placental barrier regulates the maternal–fetal interface by controlling substances transmitted to the fetus [23,24]. Both the maternal and fetal circulations access the intervillous space, allowing nutrient and waste exchange as well as limited immune surveillance [23,25,26,27,28]. Thus, the intrauterine environment is highly dependent on maternal health.

## 3. Types of Preterm Birth

While birth occurring before 37 weeks of gestation is considered preterm, the limit of viability is as low as 23 weeks in some medical centers [29]. Thus, preterm birth may be classified as: extreme (23^0/7^–27^6/7^ weeks), very early (28^0/7^–31^6/7^ weeks), moderate (32^0/7^–33^6/7^ weeks) and late (34^0/7^–36^6/7^ weeks) [2]. Multiple mechanisms may result in preterm birth, making it difficult to classify for research [16]. Despite difficulties in classifying preterm birth for research purposes, clinically it may be categorized into three different types: spontaneous preterm birth (PTB), preterm premature rupture of membranes (pPROM), and medically indicated (iatrogenic) preterm birth. Research has identified numerous risk factors for preterm labor that results in preterm deliveries. Women with extreme maternal reproductive ages (<18 or >40), obesity, behavior (use tobacco, alcohol and drug use), infections or allergic reactions, or psychosocial stress are at higher risk to have spontaneous preterm labor [2,30]. In addition, African American women and those with a lower socioeconomic status are at increased risk to deliver preterm [2,30]. Many PTB risk factors also result in DNA methylation differences. Of particular relevance, DNA methylation patterns associate with stress [31,32], diet [33,34,35,36,37], smoking [38,39,40,41,42], inflammatory cytokine levels [43] and medications [44,45,46,47].

Medically indicated or iatrogenic preterm birth results from obstetrical, fetal or medical complications requiring early delivery for treatment or resolution. Common obstetrical indications for preterm delivery include gestational diabetes and severe preeclampsia (new onset elevated blood pressure associated with inadequate fetal growth or perfusion and maternal organ damage) [48]. Both these illnesses must be treated by delivery and adjunctive therapies to completely resolve the condition [49,50,51,52]. Fetal indications for delivery may also occur without maternal pathology. For instance, fetal hydrops, a consequence of severe fetal anemia, requires blood transfusions in utero, but does not negatively impact the mother’s health. If possible iatrogenic preterm delivery occurs after achieving fetal lung maturity [53]. Finally, maternal medical disease such as a new cancer diagnosis may dictate an early delivery in order to appropriately treat a life-threatening disease without adversely impacting the fetus [54].

Premature rupture of membranes (PROM) involves a rupture of the fetal membranes prior to the onset of labor. In an uncomplicated term pregnancy, rupture of the membranes immediately precedes or occurs during labor [55], with pPROM occurring prior to 37 weeks of gestation. pPROM is characterized by activation of inflammatory mediators, mainly cytokines, chemokines and matrix metalloproteinases, that can weaken fetal membranes via proteolytic damage. The biggest risk factors for pPROM are placental abruption, infection, uterine or cervical abnormalities, and uterine over-distension [16].

Spontaneous PTB can happen either with or without rupture of membranes. Risk factors associated with PTB are similar to that of pPROM and include, but not limited to, prior preterm birth, intra-amniotic infections, stress, behavior, obesity and inter-pregnancy interval [17]. Although risk factors are well-recognized and many intervention strategies have been developed (e.g., tocolytics for contractions, antibiotics and steroids for infection/inflammation, progesterone for cervical shortening), none of these interventions have reduced the risk of PTB over three decades. Development of PTB and pPROM treatments will be substantially enhanced by a more in depth understanding of their underlying molecular mechanisms.

## 4. Proposed Mechanisms of PTB

There are many hypotheses about the mechanisms that may contribute to PTB. Some note that PTB is heritable and seek to identify the genetic risk factors [13,56,57]. Many note that the factors that increase risk of PTB and pPROM are also fundamental in maintaining a healthy pregnancy, and these hypotheses tend to focus on neuroendocrine and immune systems.

Pregnancy is a period of extensive stress, during which the hypothalamic pituitary adrenal (HPA) axis undergoes extensive changes [58,59]. Hypothalamic corticotropin-releasing hormone (CRH) is the primary regulator of pituitary release of adrenocorticotropic hormone (ACTH). In turn, ACTH stimulates the release of glucocorticoids from the adrenal cortex. Glucocorticoids provide negative feedback on the HPA axis, and inhibit at both the hypothalamic and pituitary levels. However, during pregnancy glucocorticoid production stimulates release of placental CRH [60].

The placenta releases CRH into maternal and fetal circulation in significant quantities. Placental CRH stimulates the maternal HPA axis, leading to an increase in total and free cortisol during pregnancy [61]. The HPA axis also regulates placental blood flow and influences the timing of parturition [62,63]. CRH stimulates ACTH release both from the fetal pituitary and the placenta [64,65], which in turn leads to release of cortisol from the fetal adrenal gland [66]. Progressive activation of the fetal HPA axis is important for maturation of organs such as lungs [67,68]. Furthermore, increased levels of placental CRH are associated with onset of labor [62,63].

The neuroendocrine system plays an important role in timing of parturition. It is therefore not unexpected that dysregulation of the neuroendocrine system could be involved in the mechanism of PTB, particularly since stress is a risk factor for PTB [69]. Physical and psychological stress can activate the maternal and fetal HPA axis, which increases production of placental CRH, a vital hormone for fetal maturation as well as the initiation and timing of labor [70]. For this reason, some investigators call it the “placental clock” [14,63].

The neuroendocrine and immune systems are interconnected. Acute stress has an anti-inflammatory response, though chronic mental or physical stress can lead to a pro-inflammatory state and even glucocorticoid resistance [14,71]. This is illustrated by a recent study that showed that pregnant women of low socioeconomic status are more likely to have glucocorticoid resistance and a dysregulated inflammatory response [72]. Perceived stress, cortisol, inflammation and early life socioeconomic status associate with DNA methylation differences [73]. Early life socioeconomic status may continue to impact DNA methylation in women throughout their childbearing years [74].

Over the course of a typical pregnancy, the maternal immune system is characterized by a shift from a pro-inflammatory state that is effective against intracellular pathogens such as viruses and bacteria to an anti-inflammatory state that targets extracellular pathogens using specific antibodies; however, it does so through a dynamic process [75,76,77,78,79]. The first trimester is characterized by a strong pro-inflammatory state, because of the necessity to repair the endometrium after implantation of the blastocyst and to establish placentation [80]. At the beginning of the second trimester, the maternal immune system transitions to an anti-inflammatory state to facilitate rapid fetal growth and development [80]. During the third trimester, transition to a pro-inflammatory state promotes uterine contractions and delivery of the fetus and placenta [80]. Pro-inflammatory cytokines stimulate prostaglandin and matrix metalloproteinases production, which are involved in cervical ripening, membrane rupture and uterine contractions [70,81]. Consistent with this, there is an increase in pro-inflammatory cytokines in maternal plasma and migration of leukocytes to the myometrium prior to the onset of spontaneous term labor [82,83].

Inflammation is implicated in most PTB. Various infections such as urinary tract infections, bacterial vaginosis, sexually transmitted infections, malaria and even periodontal disease have associated with PTB [84,85]. Even subclinical intrauterine infections stimulate the release of pro-inflammatory proteins that overlap with the mechanism of normal parturition. Inflammation is part of the normal signaling pathway for parturition, and a premature activation of this pathway may lead to premature labor. For example, deliberate infection of mice increases pro-inflammatory cytokines (IL-1 and TNF-alpha) and induces labor [86].

## 5. Consequences of PTB

Clinical advancements have significantly reduced the mortality rate of infants delivered preterm, but morbidity remains a substantial concern. It is unclear whether those born preterm are able to meet developmental milestones in a time frame that is comparable to their term-born peers. Even among those that do not have congenital malformations, many infants delivered preterm appear to have distinct developmental trajectories that differentiate them from term infants as they age. For example, some report that preterm infants are able to catch up in both weight and height in the first two years of life [87,88,89,90], though catch up growth could continue into childhood and adolescence [91]. Another report suggests a life-long discrepancy in height [92].

In addition to the question of whether or not preterm infants catch up, it is unclear if there are additional consequences that result from accelerated growth rates. Catch up growth in early and late infancy has been associated with obesity, cardiovascular disease, and insulin resistance in adolescents [6,7]. For example, models of maternal under-nutrition, as well as low protein and high fat diets, support epigenetic modifications and phenotypic changes in the offspring, including alterations in food preferences and cholesterol regulation [93,94,95]. Others report higher rates of behavioral and emotional problems as well as decreases in cognitive performance during childhood [5,96,97]. Similarly, studies report that children born preterm are more likely to experience slower motor, language and neurological development than children born at term [98]. Finally, children born preterm may be less likely to complete high school or seek higher education [99,100].

## 6. DNA Methylation Studies of PTB

Numerous studies report epigenetic differences associated with gestational age and growth patterns [101,102,103,104]. For example, one study reported extensive gestational age-associated DNA methylation differences even among term births and noted umbilical cord blood DNA methylations differences in genes implicated in labor and delivery [104]. However, despite extensive interest in the biological mechanisms of PTB, there are surprisingly few epigenetic studies of PTB (Table 1) [105,106,107,108]. Those that have been conducted are promising and provide insight into the mechanisms underlying PTB risk factors and consequences. 

**Table 1:** Overview of DNA methylation studies of PTB.

Tissue	Design	N	Outcome	Reference
Myometrium	Candidate gene	53	PTB	[109]
Cervical swab	LINE-1 & candidate gene	80	Gestational length	[110]
Amnion	HumanMethylation27	121	PTB & labor	[111]
Placenta	HumanMethylation27	206	Smoking & gestational age	[42]
Cord blood	Candidate gene	181	PTB & infection	[112]
Cord blood	HumanMethylation450	50	PTB & gestational age	[113]
Cord & maternal blood	LINE-1	2393	PTB	[114]
Whole blood at 19 years	Candidate gene	113	SGA	[104]
Blood spots	Candidate gene	49	Bacterial sepsis	[115]
Cord blood	Illumina Cancer Panel 1	178	Child growth	[116]
Blood spots at birth & 18 years	HumanMethylation450	24	PTB	[117]

Because of the complexity of the maternal–fetal interface and the number of tissues involved in pregnancy and delivery, it is not always clear which tissues is the most appropriate for PTB studies. For example, a recent study evaluated myometrium, the middle layer of the uterine wall that induces contractions during labor [109,118]. By comparing DNA methylation of genes that are involved in contraction, the authors sought to identify differences in PTB and term birth samples. The study categorized their 53 samples into six different delivery/labor types and found that DNA methylation of several CpG sites distinguished the groups [109]. A study by Burris and colleagues examined another maternal tissue, the cervix, which is separates the uterus and vagina. They evaluated global methylation (long interspersed nuclear elements; LINE-1) in cervical swabs collected between 16–19 weeks of gestation and reported increased LINE-1 methylation was associated with shorter gestation. In the same report, they also evaluated DNA methylation of *PTGER2* (prostaglandin E receptor 2) and reported associations with local inflammation and length of gestation [110].

Other studies focus on the role of the placenta. For example, one study examined amnion tissue, the inner layer of the fetal membranes, from 121 term and preterm deliveries [111]. This genome-wide investigation identified CpG sites that associated with both labor and PTB. The authors propose that DNA methylation changes in the amnion may participate in labor and the etiology of preterm birth, which supports the idea that DNA methylation studies can provide insight into the mechanism that contribute to causes and consequences of PTB. Similarly in a study of 206 placentas, Maccani and colleagues identified the association of DNA methylation of CpG sites in *RUNX3* (runt-related transcription factor 3) with smoking during pregnancy and lower gestational age [42].

Most preterm birth studies that examine DNA methylation use blood because of its accessibility. For example, a study using cord blood examined the association between DNA methylation of imprinted genes and both preterm birth and infection status [112]. While this study identified no association with preterm birth, they did note an association between methylation of *PLAGL1* (pleiomorphic adenoma gene-like 1) and chorioamnionitis. In contrast, a comprehensive evaluation of umbilical cord blood in African Americans by Parets and colleagues identified thousands of CpG sites across the genome that associated with PTB; the associated genes were enriched for numerous development processes [113]. This study also identified many CpG sites that were associated with PTB [117] and with gestational age in term births [102] in other studies. Replication identifies the most robust and important differences that can be targeted for therapies. Finally, a study by Burris and colleagues examined LINE-1 methylation and found that it is more heavily methylated in maternal blood in early pregnancy. They also reported that lower LINE-1 methylation levels in early pregnancy associated with increased risk of PTB while the opposite was true for umbilical cord blood [114]. While this may appear contrary to their previous result [110], DNA methylation is tissue specific so results from blood and cervical tissue cannot be directly compared. However, each tissue may provide an important window into the biological processes relevant for PTB.

Many hope that DNA methylation studies will yield biomarkers that can be used to screen for preterm birth or its risk factors. One such study explored DNA methylation to diagnose bacterial sepsis, a generalized immune response that is likely to affect children who are born preterm with low birth weight or very low birth weight [115]. The researchers examined CpG sites in the promoter of *CALCA* (calcitonin). Though the study was preliminary, they report DNA methylation differences in this region in infants with early onset sepsis and late onset sepsis that were not present in matched controls or neonates with isolated infections. Epigenetic biomarkers have been utilized in a number of diseases that primarily affect adults [119,120,121]. This promising clinical study supports the role of DNA methylation in obstetrics and neonatal clinical care.

## 7. DNA Methylation Studies of Long Term Outcomes of PTB

DNA methylation may provide insight into the long-term effects of PTB. In a comparison of children born preterm and at term, Relton and colleagues measured methylation of numerous genes at birth and reported an association with body size at approximately 9 years [116]; methylation of a CpG site in alkaline phosphatase (*ALPL*) associates with height, and the authors discuss the role of this gene in bone mineralization. Similarly, a longitudinal study comparing DNA methylation across the genome in 12 individuals born preterm to 12 born at term reported numerous methylation differences at birth. Interestingly, some of those CpG sites still distinguished preterm and term birth at 18 years of age [114]. Despite the small sample size, this is an important preliminary study that supports the stability of some epigenetic differences in those born preterm.

## 8. Recommendations for Future Studies

There are numerous challenges to incorporating DNA methylation into PTB studies as evidenced by the paucity of literature on the subject. The studies performed to date are conducted with relatively small sample sizes or limited to specific genes. While many findings have been reproducible, larger studies will identify differences of more subtle effect sizes. Another challenge involves the rates of extreme, early and late preterm inclusions as well as variations in fetal characteristics such as birth weight. For example, a study evaluated the association between DNA methylation in four genes (*IGF2*, *GNASAS*, *INSIGF* and *LEP*) and small for gestational age (SGA) among primarily preterm neonates [106]. They reported no association between DNA methylation of these genes and SGA, with only 38 SGA subjects, lack of association difficult to interpret. Efforts to limit phenotypic heterogeneity will also increase reproducibility and simplify interpretation of these studies.

Many studies conducted to date exclusively collect samples at the time of delivery. While these cross-sectional studies provide insight into differences in methylation patterns between preterm and normal-term infants, they are not well suited to illuminate the consequences of those differences. While there are numerous DNA methylation differences between PTB and term births, similar differences are observed across gestational ages [104]. Despite this, cross-sectional studies may be particularly useful for biomarker development as epigenetic patterns that predict PTB-related morbidities must be extensively evaluated. Similarly, tissues that can be obtained prior to delivery, such as maternal samples, may provide insight to the mechanism of PTB or biomarkers that are useful for identifying and monitoring those at risk for delivering preterm or other adverse pregnancy outcomes. For example, Elovitz and colleagues recently reported differences in miRNA expression in the cervical cells of women who delivered preterm compared to those who delivered at term; the authors suggested that selected miRNAs may help to identify new therapeutic targets and facilitate early screening [122]. While DNA methylation often correlates with other epigenetic modifications, evaluation of additional modifications, including miRNA, will undoubtedly provide a more complete understanding of PTB and its consequences.

To assess the potential effects of DNA methylation on spontaneous parturition, rigorous studies examining DNA methylation signatures during pregnancy are necessary. Establishment of epigenetic reference panels in multiple tissues throughout pregnancy and early development will serve as an important resource for interpreting the functional significance of PTB-associated DNA methylation differences that have already been identified. Prospective studies should be conducted to include longitudinal sampling at varying time points during pregnancy, with follow up in the child and mother following preterm delivery. The field would further benefit by inclusion of clinical samples, such as amniocentesis or cell free fetal DNA, in those reference panels. This will allow researchers to better understand the factors that promote epigenetic differences and the predictive value of those differences.
